# Hypergravity-induced malfunction was moderated by the regulation of NMDA receptors in the vestibular nucleus

**DOI:** 10.1038/s41598-021-97050-8

**Published:** 2021-08-31

**Authors:** Gyutae Kim, Kyu-Sung Kim

**Affiliations:** 1 Research Institute for Aerospace Medicine, Inha University, Incheon, Korea; 2 Department of Otolaryngology Head and Neck Surgery, Inha University Hospital, Incheon, Korea

**Keywords:** Neuroscience, Physiology

## Abstract

Gravity alteration is one of the critical environmental factors in the space, causing various abnormal behaviors related with the malfunctioned vestibular system. Due to the high plastic responses in the central vestibular system, the behavioral failures were resolved in a short period of time (in approx. 72 h). However, the plastic neurotransmission underlying the functional recovery is still elusive. To understand the neurotransmitter-induced plasticity under hypergravity, the extracellular single neuronal recording and the immunohistochemistry were conducted in the vestibular nucleus (VN). The animals were grouped as control, 24-h, 72-h, and 15-day exposing to 4G-hypergravity, and each group had two subgroups based on the origins of neuronal responses, such as canal and otolith. The averaged firing rates in VN showed no significant difference in the subgroups (canal-related: *p* > 0.105, otolith-related: *p* > 0.138). Meanwhile, the number of NMDAr was significantly changed by the exposing duration to hypergravity. The NMDAr decreased in 24 h (*p* = 1.048 × 10^–9^), and it was retrieved in 72 h and 15 days (*p* < 4.245 × 10^–5^). Apparently, the reduction and the retrieval in the number of NMDAr were synchronized with the generation and recovery of the abnormal behaviors. Thus, the plasticity to resolve the hypergravity-induced malfunctional behaviors was conducted by regulating the number of NMDAr.

## Introduction

Neurolab mission (NASA, 1993) initiated the research interests, such as balance, sensory integration, sleep, blood pressure control, and nervous system in space, and most of these topics are closely related with the function of the vestibular system. Especially, the vestibular responses were directly affected by the change of gravity, inducing the neural plasticity for the adaptation^1^. The effects of gravity sensation were various ranged from sensory balance^2,3^ to cognition^4–6^. To demonstrate the adaptation to the modified sensation, the neural plasticity in the central vestibular areas has been often assessed, and the main approaches were conducted by examining behavioral responses, neural activities, and some relevant proteins^7–9^. The essence of these assessments depended on the molecular and cellular modification, and the glutamates and their receptors were considered as the core factors because of their abundance in the brain^10,11^. As a dominant type of the excitatory neurotransmitters, the glutamates were driven mainly by ligand-gated ion channels, which provided the fundamental pathway for the glutamates^12^. Thus, the expression of the channels was an evident indicator to show the gravity-induced effects for the plasticity. Supportively, the glutamate and GABA (γ-amino-butyric acid) receptors have been known to dominantly govern most synaptic transmission in the vertebrate central nervous system^13^.

Of the glutamate-gated ion channels, α-amino-3-hydroxyl-5-methyl-4-isoxazole-propionic acid (AMPA) and *N*-methyl-d-aspartate (NMDA) receptors mainly mediate the glutamate transmission and the plasticity in the vestibular nucleus (VN)^14^. Generally, AMPA receptors (AMPAr) have been known to relate with the general transmission of the vestibular neural information^14,15^ while NMDA receptors (NMDAr) involve the neural plasticity, increasing the permeability of calcium ions (Ca^2+^)^14,16–18^. In addition, the metabotropic and the delta-2 glutamate receptors in the cerebellum have been also known as the units for the plasticity^19,20^. To assess the effects on these glutamate receptors by the gravity alteration, the changes in mRNA expression of the receptors was investigated with the expectation that the changes in the gene expression had a close relation with those in receptors^21,22^. According to the wide range of investigations on the mRNA expression from the vestibular ganglion to the cerebellum, the gravity had limited effects on the gene expression, indicating that the gravity changed the glutamatergic neurotransmission at the vestibular periphery^22^. However, these previous results rarely explained the morphological changes for the neural plasticity in the central vestibular system, and they also failed to provide the relation between the changes in mRNA expression and glutamate receptors. Meanwhile, the modeling study for the neuronal adaptation by gravity suggested that the hypergravity increased the probability of ion channel open state^1^, and the result also implied the increase in the number of activated receptors. Thus, the change in the number of the glutamate receptors possibly shows the direct effects by the gravity alteration. Here, we examined the effects of hypergravity on the alteration of the number of glutamate receptors, focusing on the NMDAr in the vestibular nucleus (VN), which has been widely known as the core receptor for the neural plasticity. Examining the alteration, the neural response to the change of gravity was demonstrated, and the neural plasticity by hypergravity was examined.

## Results

**Figure 1:**
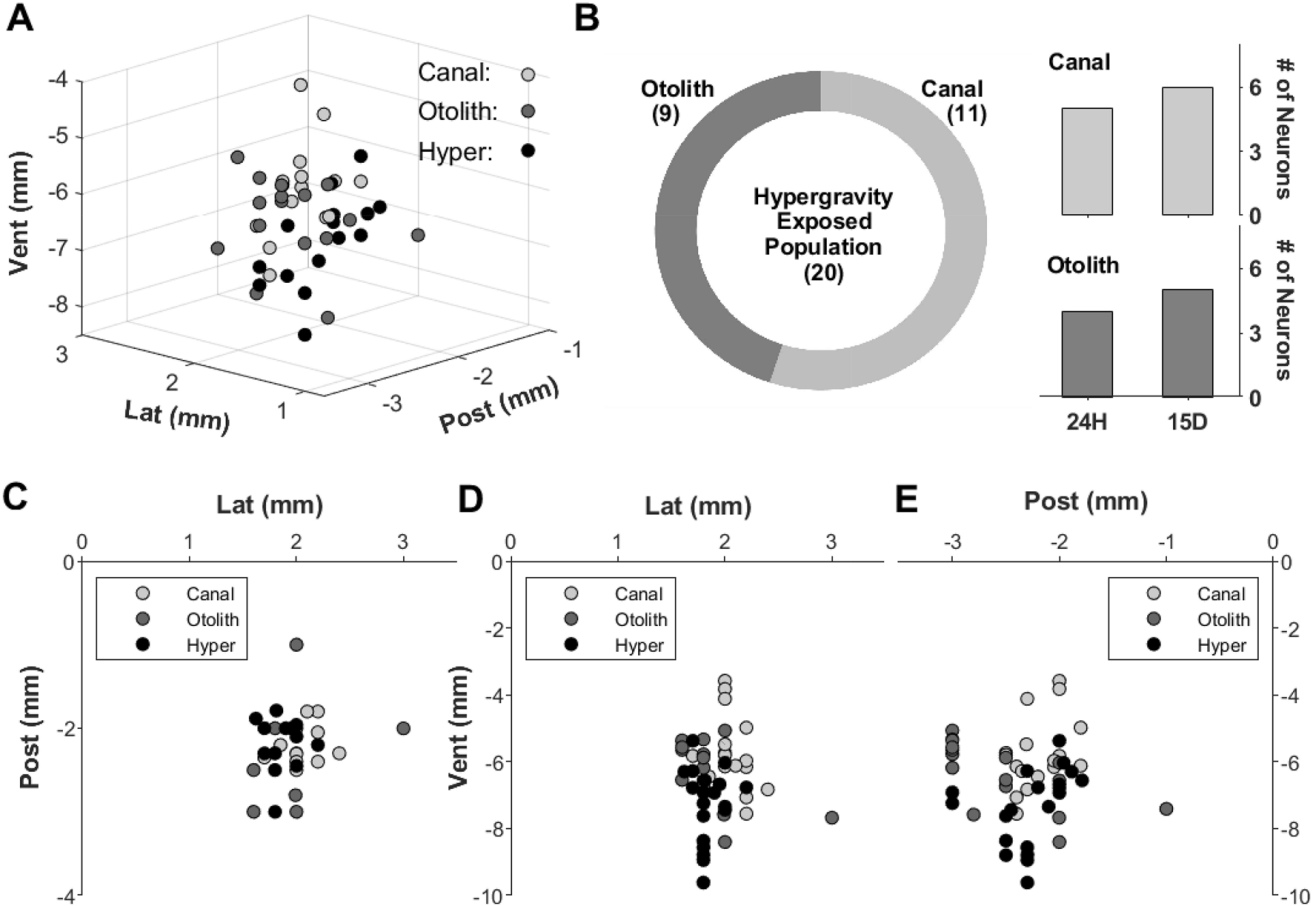
3D recording positions for extracellular neuronal activities (**A**) and the recorded population distribution, depending on the neuronal responses to kinetic stimulations (**B**). 2D recording positions were provided from different aspects of views.

Forty five animals (SD, 188-353 g, males) were used in this study, and 22 and 23 animals were used for the extracellular neuronal recording and the immunohistochemistry, respectively. Total animals for the neural recording were again divided into three groups; canal-related control (n = 8), otolith-related control (n = 7), and hypergravity-exposed groups (n = 7). As the neuronal activities from the controls, 17 canal-related and 16 otolith-related responses were obtained, and 20 (11 canal-related and 9 otolith-related) neuronal activities were recorded after the exposure to hypergravity. Again, the activities after the exposure were classified as 24-h (5 canal-related and 4 otolith-related) and 15-day (6 canal-related and 5 otolith-related) for the acute and the chronic conditions, respectively. These divided groups were summarized in Fig. 1B. To identify the recorded positions of the neuronal activities, all the positions of the recording electrode were measured, and presented in two (2D) and three dimensional (3D) spaces. The 2D recording spaces were presented as views from the top (Fig. 1C), the back (Fig. 1D), and the right side (Fig. 1E), and all combined positions in 2D were shown in Fig. 1A. The neuronal responses to the kinetic stimuli and their recording positions suggested that the neuronal activities were obtained from VN.

**Figure 2:**
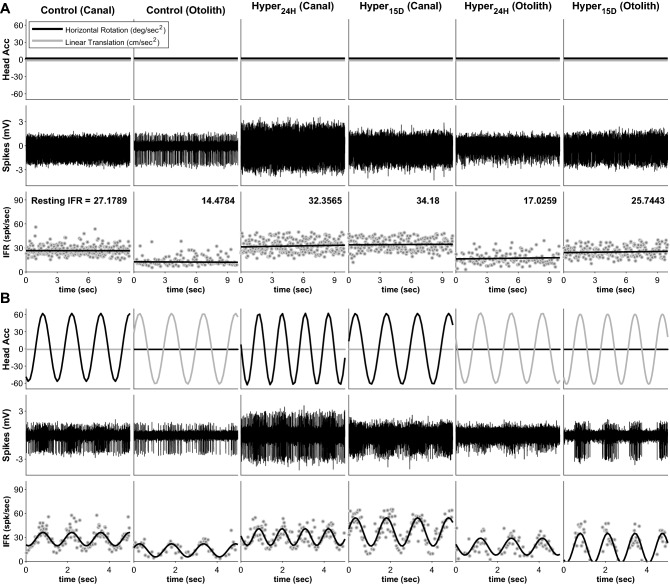
Exampled neuronal responses depending on the exposing periods to hypergravity. The averaged firing rates (FR) were obtained from the resting FR during 10-secconds long (**A**), and their responses to the given kinetic stimulations were presented (**B**). The units of head acceleration were deg/s^2^ and cm/s^2^ for horizontal head rotation (black line) and linear transmission (gray line), respectively.

**Figure 3:**
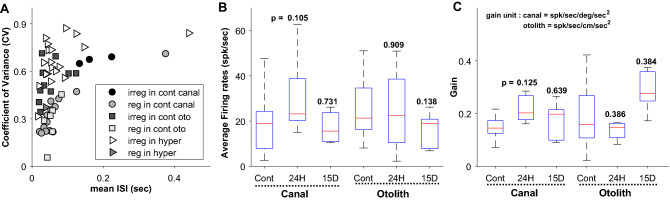
Correlation between the mean inter-spike interval and its coefficient of variance for the recorded neuronal firing regularity (**A**), the comparison of the averaged firing rates (**B**), and the gains of the neuronal responses to head acceleration among the different groups (**C**). In both static (**B**) and dynamic neuronal parameters (**C**), no statistical significance was observed.

The recorded neuronal activities were analyzed by computing the instantaneous firing rate (IFR) and its curve fitting (see Extracellular neural recording in vestibular nucleus). During the resting and the kinetic stimulation, the neuronal responses were recorded, and the representative for each categorized group was shown in Fig. 2. All resting responses were presented in Fig. 2A with the neuronal spikes and their IFR. As indicated, the head accelerations for the rotation and the linear transmission were shown in black and gray lines, respectively, and the resting responses were the neuronal activities under no head movement (Fig. 2A). Their relevant IFR (gray circle) was presented with a curve-fitted line (black line) to emphasize the neuronal responses. Based on the computation, the averaged firing rates (aFR) were estimated, and the aFRs of examples were ranged from 14.48 to 34.18 spks/s. Under the kinetic stimulation, the neuronal responses correlated to the head movement (Fig. 2B). Depending on the type of stimulation, the recorded neuronal responses were classified as a canal-related or an otolith-related activity. To identify the characteristics of the vestibular neurons, the discharge regularity was computed, and examined if there was any skewed number in two groups; regular and irregular discharging groups (Fig. 3A). In summary, all neurons were categorized as 21 regular and 32 irregular units, and there was no statistical skewness to any groups (*p* = 0.106). The comparison among the canal-related and the otolith-related groups showed no significance (Fig. 3B). Of the canal-related groups, the aFRs were 18.92 (STD = ± 12.10), 30.74 (± 18.73), and 17.09 (± 6.67) spiks/s for control, 24-h, and 15-day exposure, respectively, but there was no significance (*p* > 0.105). A similar result was obtained from the otolith-related group (25.44 ± 12.98, 24.51 ± 20.27, and 15.82 ± 8.19 for control, 24-h, and 15-day, respectively) with no significance (*p* > 0.138). The same consequence was maintained based on the dynamic neuronal response based on the neuronal modulation during the head rotation or the linear transmission. According to the analyzed gain of the neuronal response (spk/s) to the head acceleration (deg/s^2^ or cm/s^2^), no statistical significance was observed in both groups, canal-related (*p* > 0.12) and otolith-related neurons (*p* > 0.38) (Fig. 3C).

**Figure 4:**
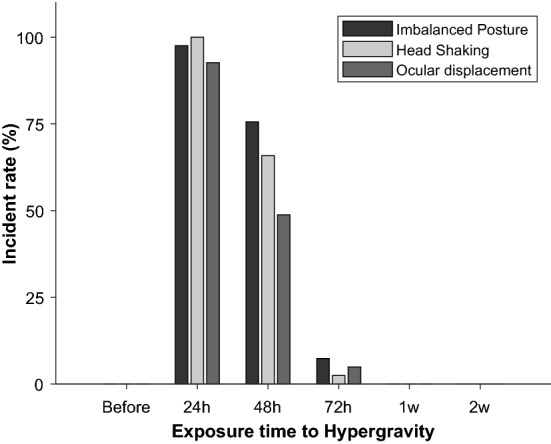
Incident rate of the malfunctioned behaviors after exposing to hypergravity. The behaviors were identified related with the general vestibular function, such as postural balance, head position maintenance, and ocular displacement.

Unlike the neuronal activities, there were notable behavioral responses to hypergravity, depending on the time of exposure. The observable behaviors were unstable posture, shaking head position, and ocular displacement (see Behavioral responses to hypergravity). These responses lasted for several days, and typically disappeared in approx. 72 h. The incident rate of the abnormal behaviors continuously decreased under ongoing stimulation, and it indicated the vestibular malfunction was the most active at 24 h later (Fig. 4). Since reaching the peak of abnormal conditions, the malfunctioned behaviors became normal, suggesting the neural plasticity which led to the functional correction in the central vestibular system. To examine the plasticity under ongoing hypergravity, the numbers of AMPAr and NMDAr were estimated, which provided the pathways of the core neurotransmitters. Due to their core roles in the neural plasticity, the glutamate receptors were widely estimated to understand the synaptic plasticity^14–18^.

**Figure 5:**
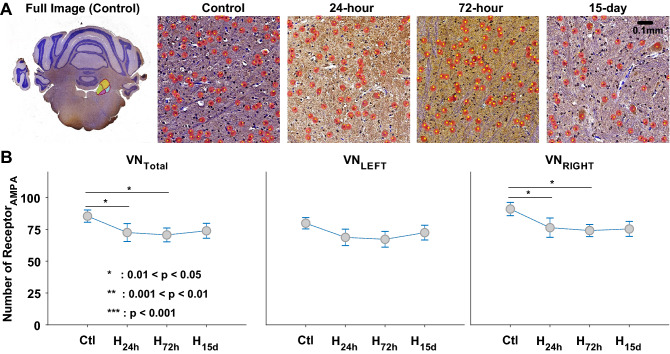
A full coronal image of the brain with the position of vestibular nucleus (VN), and the detected AMPA receptors (AMPAr) in control, 24-h, 72-h, and 15-day (**A**). Total number of AMPAr in overall, left, and right sides of VN (**B**). (*0.01 < *p* < 0.05, **0.001 < *p* < 0.01, ***p < 0.001).

**Figure 6:**
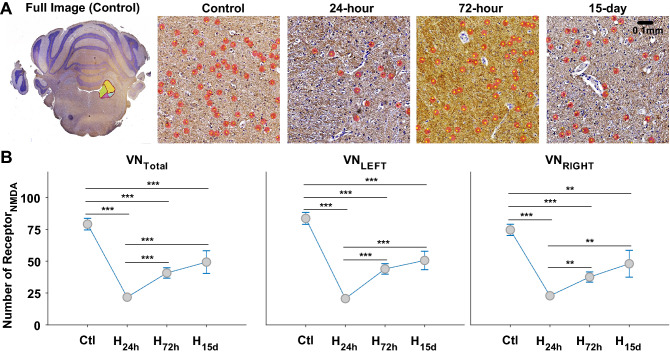
A full coronal image of the brain with the position of vestibular nucleus (VN), and the detected NMDA receptors (NMDAr) in control, 24-h, 72-h, and 15-day (**A**). Total number of NMDAr in overall, left, and right sides of VN (**B**). The significance was symbolized as done in Fig. 5 (*0.01 < *p* < 0.05, **0.001 < *p* < 0.01, ***p < 0.001).

Both types of receptors were counted using 10, 4, 4, and 5 animals for control, 24-h, 72-h, and 15-day, respectively. The numbers of AMPAr and NMDAr were counted in the whole VN with no specific separation of its sub-divisions, such as lateral, medial, and spinal VNs. The full coronal images of control were presented to specify the location of VN, and the representative sub-images for control, 24-h, 72-h, and 15-day were displayed with the detected receptors (red circles with a yellow dot) (Figs. 5A, 6A). On the full images, the sub-divisions of VN were presented in colors, and the general square dimension was approximately between 1.93 and 2.04 mm. Thus, 4–5 sub-images were used to count the total receptors under each condition. The total number of AMPAr decreased after 24-h exposure to hypergravity with significance (*p* = 0.043), but there was little recovery in 72-h and 15-day exposure (*p* > 0.051) (Fig. 5B). The changes in left and right VNs implied a similar tendency with no statistical significance (*p* > 0.094). On the other hand, the total number of NMDAr dramatically decreased after 24-h exposure (*p* = 1.048 × 10^–9^), and its recovery was also noticeable in 72 h and 15 days later (*p* < 4.245 × 10^–5^) (Fig. 6). Again, the tendency of the change was also similar in the left and the right VNs, and the alteration in the number of NMDAr was significant (*p* < 0.016). The examination on the number of glutamate receptors resulted in the significant change in the number of NMDAr under the ongoing hypergravity (Fig. 6B), and it suggested a plasticity-based link between the recoveries in behavioral responses and the number of NMDAr.

## Discussion

A neuron maintains a polarized condition during the resting phase, and its membrane potential undergoes several different stages, such as depolarization, repolarization, and hyperpolarization, to generate a neuronal spike. After proceeding these stages, the potential comes back again to the polarized condition which was its initial state. During hypergravity, a neuron was hyperpolarized, and it made its rate of action potential (AP) reduced^1,23^. Unlike the previous studies, our extracellular neuronal recording showed no significant difference in the AP rate. A possible reason was the time delay between the gravitational stimulation and the neural recording. All neural recordings were conducted after the exposure to hypergravity (see Extracellular neural recording in vestibular nucleus), but it was carried out within 2 h after completing the exposure. Although the gravitational effects on the neuronal activities were expected within the relatively short period of time, the results indicated that the gravitational effects on the AP rate were rapidly resolved no matter how long the neuron was exposed to hypergraity (Fig. 3B). The same consequence was sustained by the dynamic neuronal activities (Fig. 3C). However, some abnormal behaviors implied that the gravitational effects were preserved after completing the exposure, which was especially in approx. 72 h (Fig. 4). Moreover, the disappearance of the malfunctioned responses suggested the high possibility of neuroplasticity in the vestibular system (Fig. 4).

To identify the plasticity underlying the behavioral recovery, the neurochemical changes based on the number of glutamate receptors was examined. The abundant glutamate is known to excite the nerve cells, and the excitation lasts up to the cellular death, so called excitotoxicity^10^. To prevent the cellular apoptosis or necrosis, the glutamate receptor controls the concentration of glutamate in the extracellular fluid as well as it provides the pathway for glutamate as the core glutamate transporting system^24–26^. Under the gravitational alteration, the glutamate receptors involved the neural plasticity through the modulation of glutamate level, and it resulted in the molecular adaptation^27,28^. It has been known that the gravitational alteration leads to the morphological and genetic changes under both microgravity^29,30^ and hypergravity^22,31^. Especially, the changing gravity is highly considered as a critical environmental factor to cause the neuroplasticity in the vestibular system^32^, and the induced plastic responses were initiated in VN as demonstrated in the previous studies. This finding was also supported by the outcomes that hypergravity reduced the mRNA expression of NMDAr in VN^22^.

Current results provided two valid messages. First, the glutamate receptors involved the plastic mechanism underlying the behavioral recovery, and the NMDAr took a superior position to the AMPAr during the recovery. Both types of receptors in VN are physiologically and pharmacologically important in synaptic plasticity as well as the glutamate neurotransmission^33^, but the NMDAr are more influential on the long-term neuromodulation because of high Ca^2+^ permeability^34^. Our current data supported these previous studies, showing that the NMDAr was mostly affected by hypergravity with significance, and there was no observable relevance between the gravity alteration and AMPAr. The outcome was also the evidence to explain the behavioral changes through the long period of recovering time. Under hypergravity, the neural information of uvula/nodulus was projected on VN by increasing the activities of the glutamate receptors, and it resulted in the cerebellar inhibition for the motor control^22^. Eventually, the serial process suppressed the regulation of glutamate receptors on the post-synapse of the peripheral vestibular system, and it interrupted the transmission from the vestibular hair cells to its relevant neurons^22^. Thus, the enhanced abnormality in the behavioral responses was closely related with the changes in the number of glutamate receptors, specifically NMDAr. Second, therefore, the hypergravity-induced behavioral malfunction was resolved as the number of NMDAr was restored. Under hypergravity, the glutamate release reduced by two times more than the normal condition, and the consequence was observed only in the Ca^2+^-dependent glutamate^35^. Considering the receptors provided the primary pathway for the glutamic transmission, the reduced glutamate release was possibly linked to the decrease in the number of NMDAr, which had a high Ca^2+^ permeability. The increasing number of NMDAr during the behavioral recovery was regulated based on homeostatic mechanism^13,36^. Again, the high permeable Ca^2+^ influx by NMDAr was essential for the homeostasis, and the reduced number of NMDAr advanced the RNA reforming of NMDAr subunit, which resulted in the homeostatic regulation^36^. Thus, the failure of NMDAr regulation in VN directly contributed to some malfunctional behaviors, which required the core vestibular functions.

## Methods

Rodents (SD rats, male) were used for this study, and all procedures and principles of laboratory animal care were approved by the Animal Ethics Committee at Inha University (INHA 190107-611). In addition, the current study was reported in accordance with ARRIVE guidelines, and all methods were conducted in accordance with relevant guidelines and regulations.

### Animal preparation and hypergravity

Naïve animals were exposed to hypergravity, which was generated by a manufactured apparatus, and the overall structure was similar as those in some previous studies^13,19^. In brief, the apparatus was composed of two components with an operation and a control. These components were set separately, and the operational component was surrounded by the iron fences for the experimental safety. The operational component had two arms at 180 degrees, connecting to the centered spindle. At the end of each arm, an animal cage (a regular hexahedron with 500 mm) was attached, and up to four animals were placed in each cage during the exposure. For the balance of the arms, similar weights of animals were loaded in both cages, and the balance was maintained until the experiment was terminated. Animals were exposed to hypergravity (4G) in different durations, and the exposing durations were set as 24 h (H_24h_) and 72 h (H_72h_) for the acute and 15 days (H_15d_) for the chronic effects. During the exposure, the animals were provided with food and water, and an extra resting period (approx. 40 min) was given to the animals under the 72-h and the 15-day exposure in daily basis. Also, all animals were stayed under a 12:12 h light–dark cycle during both exposed and unexposed to hypergravity.

### Behavioral responses to hypergravity

Three types of behaviors were observed, such as postural balance, head control, and ocular position, all of which were closely related with the general vestibular functions^1,3^. The postural balance was determined by the tilted body position w/o motion. If the vestibular system was malfunctioned, the posture was biased to a side, and it resulted in the unbalanced body posture. The head control was identified as the head shaking, which rolled to clockwise and counter-clockwise directions. Generally, the head shaking was noticed when the head was apart from the floor, and the animals made their heads touched on the floor to stop the abnormal head movement. The ocular displacement was identified as the skewed ocular position to backward direction. Comparing to the ocular displacement before the exposure, the ocular position was switched to the backward direction, and it was maintained until the central malfunction was resolved. The hypergravity-induced behaviors were presented by using an incident rate which was the percentage of population with the malfunctioned behaviors (Eq. 1).

1 $${R}_{i}=100\times \frac{{n}_{malfunc}}{{n}_{total}}$$

where the incident rate (*R*_*i*_) was the percentage of malfunctioned animals (*n*_*malfunc*_) in the total population (*n*_*total*_). Thus, the incident rate was 100% if all animals showed a malfunctioned behavior. The behaviors were examined during the resting period, which was daily provided to the animals for food and water for approx. 40 min (see Animal preparation and hypergravity). The animals’ behaviors for 24-h exposure to hypergravity were examined within the similar time for a fair comparison.

### Extracellular neural recording in vestibular nucleus

Single extracellular neural recording was conducted in the vestibular nucleus (VN) before and after hypergravity, and its analysis was performed offline using a custom-made software (MATLAB 9.6, MathWorks, US). Once the exposure to hypergravity had been completed, the neural recording was performed under the animal’s anesthesis, which was carried by the injection of the solution (1 ml/kg ketamine and 0.33 ml/kg xylazine solution). The time delay from the exposure to hypergravity to the neural recording was no more than 2 h. After anesthetized, the animal’s head was fixed in the motorized stereotaxic apparatus (NEUROSTAR, Germany). The scalp was surgically removed to expose the superior surface of the skull, and the lambda was designated as the center. For a recording electrode (5MΩ, A-M system, US), a hole (2.0 mm diameter) was opened on the skull at a specific location (3.0 mm posterior and 2.0 mm lateral away from the center). All neuronal activities were continuously tested by the kinetic stimuli, such as a horizontal rotation and the linear translation following the x-axis (ipsi- and contra-lateral), which were directly related with the neural activities in VN. All neuronal signals were filtered (bandpass 0.5–3 kHz) and recorded at a sampling rate of 40 kHz using OmniPlex D system (Plexon, US). Three-dimensional (3D) location of each neuronal activity was evaluated based on the location of the recording electrode in offline, and their instantaneous firing rates (IFR) were presented to identify the correlation to the given stimulation. IFR was calculated by the reciprocal number of inter-spike interval (ISI) which was the time interval between two consecutive neuronal spikes. Once obtaining IFR (gray dots), a curve fitting (black line) was applied on the IFR to find the resting potential (Fig. 2A,B, bottoms). The equation for the curve fitting was constructed based on a sinusoidal wave form with a constant, which was used as the value for the resting potential (Eq. 2).

2 $${C}_{fit}=a\cdot \mathrm{sin}(2\times \pi \times f\times t+b)+c$$

where curve fitting (*C*_*fit*_) was a general sinusoidal function with amplitude (*a*), phase (*b*), and constant (*c*). For the IFR analysis, the neuronal spikes for 10 s were used. To assess a dynamic neuronal response, the gain of the neuronal response to the applied kinetic stimulation was computed (Eq. 3). The gain of each control group was compared with that of 24-h and 15-day exposure groups.

3 $${G}_{resp}=\frac{{Amp}_{nm}}{{Acc}_{Head}}$$

where a neuronal gain (*G*_*resp*_) was the ratio between the amplitude of neuronal modulation (*Amp*_*nm*_) and the applied head acceleration (*Acc*_*Head*_). The units of gain were spk/s/deg/s^2^ and spk/s/cm/s^2^ for the canal-related and the otolith-related neurons, respectively.

### Immunohistochemistry (IHC) and image requisition

Brains were surgically removed from the animals immediately (less than 20 min) after the termination of exposure to hypergravity. Following the fixation in 4% formaldehyde solution, the portion containing the vestibular nucleus (VN) was separated from the fixated brain, and it was prepared for a paraffin block section. The block embedded the tissue was cut with 3-µm-thick, and the section was transferred on a slide for antibody staining. The staining was performed to visualize AMPAr and NMDAr targeting their subtypes of GluR1 (Sigma-Aldrich, US) and NR1 (Sigma-Aldrich, US), respectively. The antigen retrieval solutions were Tris–EDTA based (pH 9.0, 95 °C, 50 min) for GluR1 and citrate solution (pH 6.0, 95 °C, 50 min) for NR1. After cooling and rinsing the sections, 3% hydrogen peroxide solution was applied to block endogenous peroxidase activity. Then, the prepared solutions for the subtypes were applied, and they were stored at 4 °C overnight. Once completing the visualization of subtypes, their images were obtained by a motorized microscope (Olympus, Japan). All images were basically obtained in a magnification of 400 times (ocular: × 10 and objective: × 40) of the actual size.

### Assessment of glutamate receptors in VN

All images were proceeded to count two different kinds of targets, AMPA and NMDA receptors, and their images were separately prepared. Before counting the number of receptors, the area of VN was identified in the images using a reference. VN was generally located at the distance ranged from 10.04 to 12.72 mm to the posterior direction from the bregma, with a width (0.9–2.2 mm) and a height (0.5–1.7 mm) in the coronal sections of the brain^37^. Once confirming VN, an image was split into multiple sub-images (2048 × 2048 pixels), and the sub-images at the area of VN were collected for the receptor counting. For a color contrast to detect the targets, each sub-image was re-adjusted using the ranged color histogram (140–205). The number of targeted glutamate receptors was manually assessed by several independent repetition to avoid miscounting the receptors.
